# Microsatellites Versus Genome-Wide SNPs Data for Pedigree Reconstruction in Twin Simmental Crossbred Cattle

**DOI:** 10.3390/ani16172780

**Published:** 2026-09-04

**Authors:** Xinyu Chen, Shuai Gao, Jianbo Li, Yang Luo, Baizhong Zhang, Fang He, Chuzhao Lei, Naqash Khan, Kangle Yi

**Affiliations:** 1 Yuelushan Laboratory, Changsha 410128, China; chenxinyu2003@nwafu.edu.cn (X.C.); gaoshuai32@hunaas.cn (S.G.); ljbljb12@163.com (J.L.); xinhelu509@163.com (Y.L.); zhangbz6181@163.com (B.Z.); hefang2025@hunaas.cn (F.H.); 2 Hunan Institute of Animal and Veterinary Science, Changsha 410000, China; 3 Key Laboratory of Animal Genetics, Breeding and Reproduction of Shaanxi Province, College of Animal Science and Technology, Northwest A&F University, Yangling 712100, China; leichuzhao1118@126.com (C.L.); naqashkhan2025@nwafu.edu.cn (N.K.)

**Keywords:** pedigree reconstruction, microsatellite, SNP, neighbor-joining, cattle, twinning, admixture

## Abstract

Accurate pedigree reconstruction is critical for genetic evaluation in admixed cattle populations, yet the relative performance of microsatellite and genome-wide SNP markers in twin-rich herds with incomplete pedigree records remains unclear. We compared 12 ISAG-recommended microsatellite markers with whole-genome SNP data for dam–calf assignment in a Simmental crossbred population (*n* = 43, 13 dam–calf groups) from southern China. Twin zygosity was determined from SNP identity-by-descent (PI_HAT) values: nine calf pairs were dizygotic, one pair was monozygotic (20A/21A), and one adult pair was composed of dizygotic twin sisters (31A/34A). Admixture analysis at K = 3 revealed ancestry proportions of 50.7% European taurine, 28.9% Chinese indicine and 20.4% East Asian taurine. The SNP-based neighbor-joining tree correctly recovered 12 of 13 groups (92.3%, 95% CI: 64.0–99.8%), whereas the microsatellite-based tree recovered 11 (84.6%, 95% CI: 54.6–98.1%); the difference was not statistically significant (exact McNemar test, *p* = 1.0). Locus INRA023 was monomorphic (PIC = 0), reducing the effective number of markers to 11. These results indicate that genome-wide SNPs show a favourable trend in accuracy and are less prone to false-positive clustering than a standard microsatellite panel in admixed, twin-rich cattle populations.

## Simple Summary

This study compared two DNA-based methods for determining mother-calf relationships in a herd of crossbred cattle with a high rate of twinning. One method used 12 standard microsatellite markers, and the other used thousands of genome-wide SNP markers. The SNP method correctly identified 92.3% of known mother-calf groups, while the microsatellite method identified only 84.6% and produced several incorrect groupings. One of the 12 microsatellite markers was completely uninformative in this population, contributing to the lower accuracy. Among the calves, nine pairs were dizygotic twins, one pair was monozygotic, and an additional pair of adult cows was identified as dizygotic twin sisters. These results indicate that genome-wide SNPs show a favourable trend in accuracy and are less prone to false-positive clustering for pedigree reconstruction in genetically diverse cattle populations, especially when pedigree records are incomplete.

## 1. Introduction

Frequent twinning complicates dam–calf identification, especially in smallholder production systems where detailed pedigree records are often unavailable [1]. Simmental crossbred cattle in southern China have a complex genetic background due to introgression from both *Bos indicus* and *Bos taurus* lineages [2,3]. Microsatellite markers have been widely used for parentage verification in cattle populations [4,5,6]; however, their performance in admixed, twin-rich populations with incomplete pedigree records has not been systematically evaluated against genome-wide SNP data. Furthermore, while previous studies have compared microsatellite and SNP panels in purebred or well-documented herds [7,8], the joint interplay of mixed ancestry, frequent twinning and incomplete farm records remains underexplored. From a molecular marker perspective, microsatellites are particularly susceptible to allele dropout, null alleles, and size homoplasy in genetically diverse populations, which may compromise their resolution for pedigree inference.

Twinning in cattle can involve either monozygotic (genetically identical) or dizygotic (fraternal) twins. In pedigree reconstruction, dizygotic twins pose a greater challenge as they share the same expected relatedness to parents as ordinary full-siblings, yet farm records may not always distinguish between the two types. Despite the practical importance of twin-typing for genetic evaluation, few studies have explicitly accounted for zygosity when comparing marker-based pedigree methods.

The comparison was not intended as an equal-marker-count test but as a comparison of two practical genotyping strategies currently used in cattle parentage testing: the standard ISAG microsatellite panel and genome-wide SNP data.

Here, we compared 12 ISAG-recommended microsatellite markers with genome-wide SNP data for recovering dam–calf pairs in a twin-rich Simmental crossbred herd from Hunan Province, southern China. We explicitly determined twin zygosity, incorporated statistical comparisons of method performance, and assessed the impact of population admixture and marker informativeness on the accuracy of both methods.

## 2. Materials and Methods

### 2.1. Animals and Samples

A total of 43 Simmental crossbred cattle were sampled from farms in Hunan Province, China, including 17 adult females and 26 calves. Among the 17 dams, 13 were confirmed as the true mothers of sampled calves based on the combined evidence of farm records and pairwise identity-by-descent (IBD) analysis from SNP data (PI_HAT, defined as the proportion of the genome shared identically by descent between two individuals). This represented 13 known dam–calf groups with 23 confirmed dam–calf pairs. Among the remaining adult cows, 25A, 26A, and 30A were included as background individuals with no genetic relationship to any calf. Individual 34A, although classified as a background dam with respect to calving records, was subsequently identified as the dizygotic twin sister of 31A based on SNP PI_HAT analysis and therefore played a secondary role in one of the ambiguous pedigree clusters. Of the 26 calves, 23 were confirmed as true offspring of the 13 dams (comprising ten complete twin pairs and three singletons), while three calves (11A, 18A, 19A) were excluded from their originally recorded dams based on preliminary microsatellite screening and were retained as additional individuals in the analysis. The use of microsatellite-based pre-screening was adopted to identify gross mismatches rapidly and cost-effectively before performing whole-genome sequencing, following the standard practice in cattle parentage testing. All calves in the 13 confirmed groups were typed for zygosity based on pairwise PI_HAT values from SNP data: calves with PI_HAT ≈ 0.5 were classified as dizygotic twins (nine pairs), and the pair with PI_HAT ≈ 1.0 (20A/21A) was classified as monozygotic twins. Additionally, adult cows 31A and 34A were identified as a dizygotic twin-sister pair based on their genetic relatedness. Detailed sample information is provided in Table S1.

Complete pedigree records, including sire and maternal-grandsire information, were not available for this population. All samples were collected under the approval of the Experimental Animal Management Committee (EAMC) of Northwest A&F University (Approval No. 2011–31101684, 1 January 2011).

### 2.2. Genomic DNA Extraction and Sequencing

Genomic DNA was extracted from blood samples using a standard phenol-chloroform protocol [9]. Whole-genome resequencing was performed on an Illumina NovaSeq 6000 platform (Illumina Inc., San Diego, CA, USA) with 2 × 150 bp paired-end reads, yielding an average depth of 12.7×. Clean reads were aligned to the ARS-UCD1.2 reference genome using BWA-MEM (v0.7.13-r1126) [10,11] and processed with SAMtools (v1.9) [12]. Variant calling was conducted using GATK (v3.8) [13,14], and high-quality biallelic SNPs were retained after hard filtering: Quality by Depth (QD) < 2.0, Fisher Strand bias (FS) > 60.0, Root Mean Square Mapping Quality (MQ) < 40.0, Mapping Quality Rank Sum Test (MQRankSum) < −12.5, Read Position Rank Sum Test (ReadPosRankSum) < −8.0, Strand Odds Ratio (SOR) > 3.0, and mean sequencing depth per site within 1/3 to 3-fold of the average genome depth. SNPs failing any of these criteria were excluded from further analysis. During the VCF-to-PLINK conversion using VCFtools (0.1.17), SNPs with minor allele frequency (MAF) < 0.05 were removed. After these quality control procedures, 23,684,319 SNPs were retained for subsequent analyses. LD pruning was then performed using PLINK v1.9 [15] with the parameters –indep-pairwise 50 10 0.1 (a 50-SNP sliding window with a 10-SNP step, removing one SNP from each pair with r^2^ > 0.1), yielding a final set of 654,363 SNPs for downstream phylogenetic analyses. This parameter combination is widely adopted in cattle genomic studies to reduce marker redundancy while retaining sufficient information for relationship inference and population structure analysis.

For microsatellite analysis, 12 ISAG core markers [16] were amplified using fluorescently labelled primers (Sangon Biotech Co., Ltd., Shanghai, China) (Table S2). Fragment analysis was performed on an ABI 3730xl sequencer (Applied Biosystems, Foster City, CA, USA) using GeneMapper v5.0 software (Applied Biosystems, Foster City, CA, USA).

### 2.3. Population Structure Analysis

We characterized population structure using ADMIXTURE v1.3.0 [17]. Ancestry proportions were estimated for K = 1–7 (where K represents the assumed number of ancestral populations) using five-fold cross-validation. Reference populations included 93 individuals from nine breeds: Chinese indicine (Leiqiong, *n* = 13; Weizhou, *n* = 17), Indian indicine (Sahiwal, *n* = 10; Tharparkar, *n* = 7; Brahman, *n* = 4), East Asian taurine (Hanwoo, *n* = 15), Eurasian taurine (Simmental, *n* = 8; Holstein, *n* = 7), and European taurine (Angus, *n* = 12), with whole-genome sequencing data obtained from public databases [2,3]. The breed-level ancestry assignments of the reference populations allowed the three K = 3 components to be identified as European taurine, East Asian taurine, and Chinese indicine lineages. Principal component analysis was performed with EIGENSOFT v5.0 [18].

### 2.4. Pedigree Reconstruction

Pedigree reconstruction was conducted using a distance-based clustering approach [19,20]. For SNP data, pairwise genetic distances were calculated using the genome-wide identity-by-state (IBS) matrix implemented in PLINK v1.9 [15]. To provide phylogenetic context, we included the same reference panel of 93 individuals described above. A neighbor-joining (NJ) tree was then constructed [21]. For microsatellite data, Bruvo distances were calculated using the R package poppr (v2.9.6) [22] and an NJ tree was constructed using the same approach. Recovery of known dam–calf pairs was assessed by inspecting the NJ tree topology. Successful recovery was defined as all calves from a known dam forming an exclusive monophyletic clade together with their true dam, with no unrelated individuals nested within the clade [21,23].

To further validate the SNP-based reconstructions, pairwise identity-by-descent (IBD) was estimated using PLINK v1.9 [15].

### 2.5. Statistical Comparison of Methods

A two-sided exact McNemar test was used to compare the paired recovery outcomes of the SNP- and microsatellite-based NJ trees across the 13 dam–calf groups. Exact 95% confidence intervals for the recovery rates were calculated using the Clopper–Pearson method. An exact test was used instead of the asymptotic chi-squared version due to the small number of paired groups, which renders the chi-squared approximation unreliable.

## 3. Results

### 3.1. Population Admixture and Genetic Diversity

**Figure 1:**
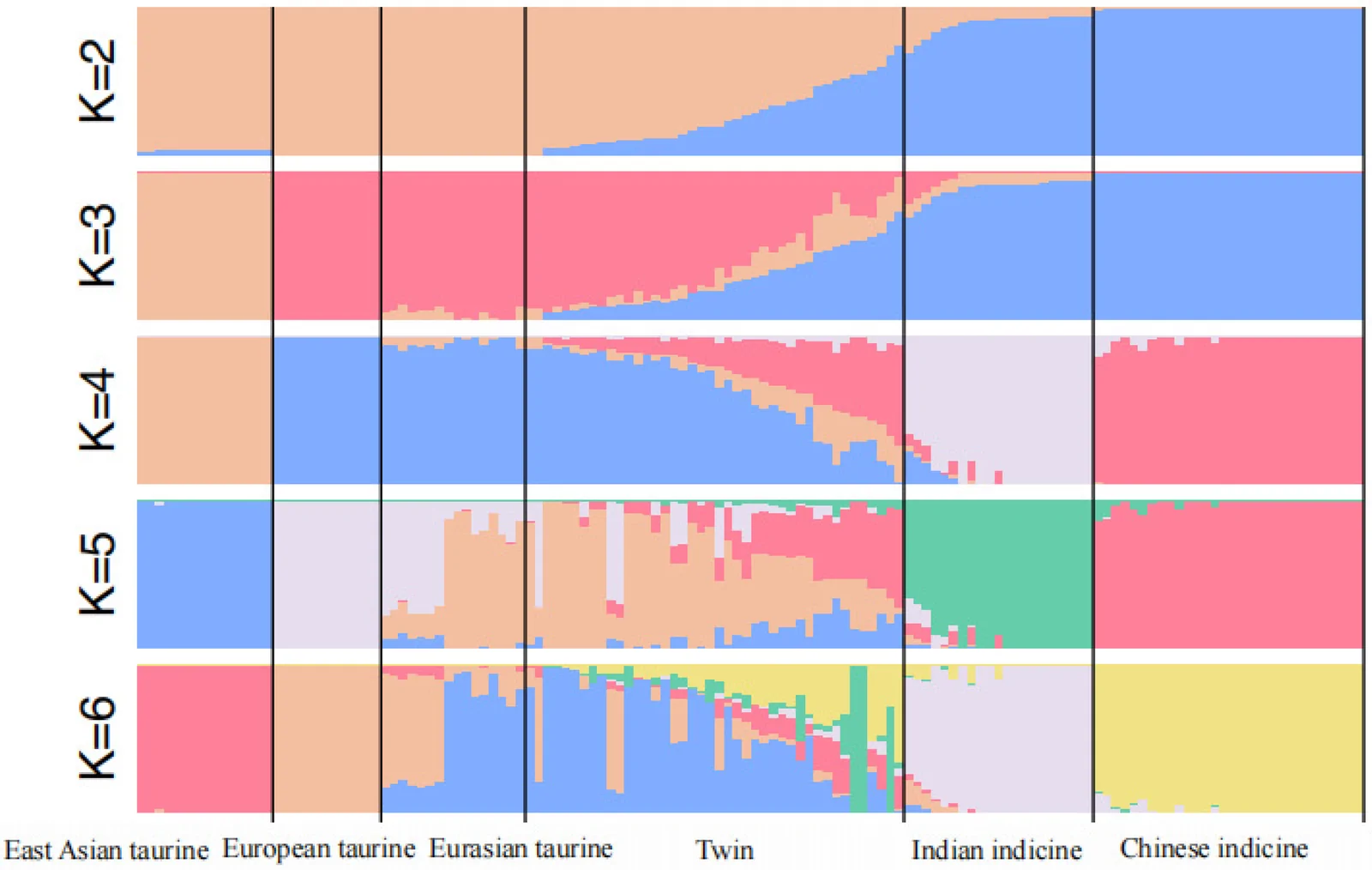
Ancestry proportions estimated by ADMIXTURE at K = 3 for the 43 Simmental crossbred cattle and 93 reference individuals from nine breeds. Each vertical bar represents an individual, and colors indicate the proportional membership in each of the three inferred ancestry components (European taurine, East Asian taurine, and Chinese indicine).

Cross-validation error in the admixture analysis decreased sharply from 0.687 at K = 1 to 0.513 at K = 2 and remained relatively stable across K = 2 to K = 7 (0.513–0.504), with a slight minimum at K = 5 (0.503). Given the absence of a clear inflection point and the biological interpretability of the three known cattle lineages (European taurine, East Asian taurine, and Chinese indicine), we present results at K = 3. The three ancestry components at K = 3 were assigned to European taurine, East Asian taurine, and Chinese indicine lineages based on the predominant ancestry of the reference populations included in the analysis. At K = 3, the average ancestry proportions in the Hunan Simmental crossbred population were 50.7% European taurine, 28.9% Chinese indicine and 20.4% East Asian taurine, with high individual variation. For example, individuals 36A and 37A exhibited >93% European taurine ancestry, while individuals 6A, 17A and 25A showed >49% Chinese indicine ancestry. These results confirm the complex hybrid origin and high genetic diversity of this population (Figure 1).

**Figure 2:**
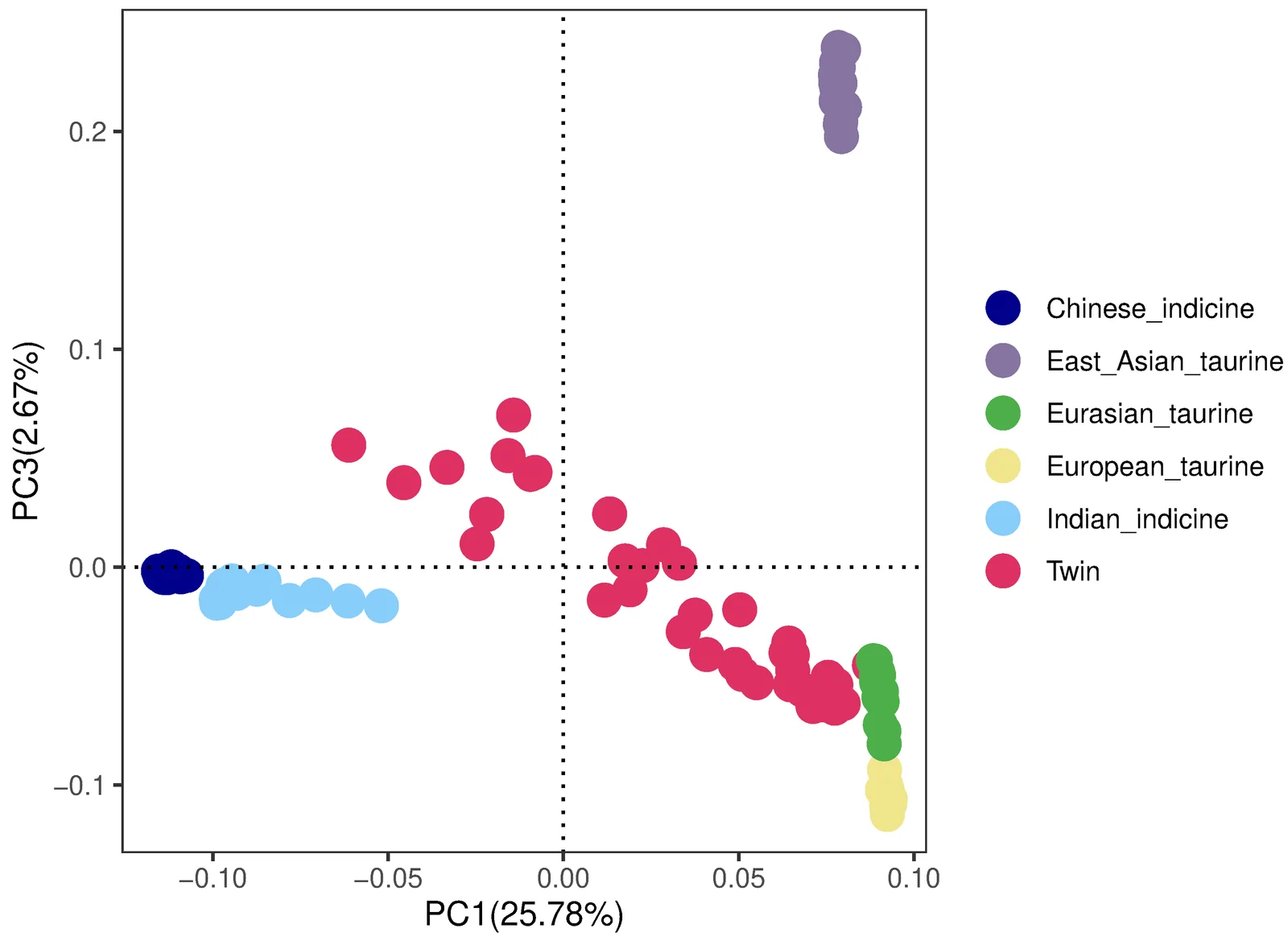
Principal component analysis (PCA) of the 43 Simmental crossbred cattle and 93 reference individuals. PC1 (25.78% of variance) separates *Bos indicus* from *Bos taurus* lineages; PC3 (2.67% of variance) distinguishes East Asian taurine from European and Eurasian taurine populations. The dashed lines indicate PC1 = 0 and PC3 = 0 reference lines. The Hunan Simmental crossbred samples are distributed along the genetic cline between *Bos indicus* and *Bos taurus*, consistent with their admixed ancestry.

Principal component analysis corroborated these findings (Figure 2). The first principal component (PC1, explaining 25.78% of the genetic variation) clearly separated *Bos indicus* from *Bos taurus* lineages. The Hunan Simmental crossbred cattle were distributed along a genetic cline between these two major groups, consistent with their hybrid ancestry. The third principal component (PC3, explaining 2.67% of the variation) further resolved the substructure within *Bos taurus*, distinguishing East Asian taurine from European and Eurasian taurine populations.

Twin zygosity analysis based on SNP PI_HAT values confirmed that the twins 20A/21A were monozygotic (PI_HAT ≈ 1.0), while the other nine calf twin pairs were dizygotic (PI_HAT ≈ 0.5). Additionally, adult cows 31A and 34A were identified as a dizygotic twin-sister pair.

### 3.2. SNP-Based Pedigree Reconstruction

**Figure 3:**
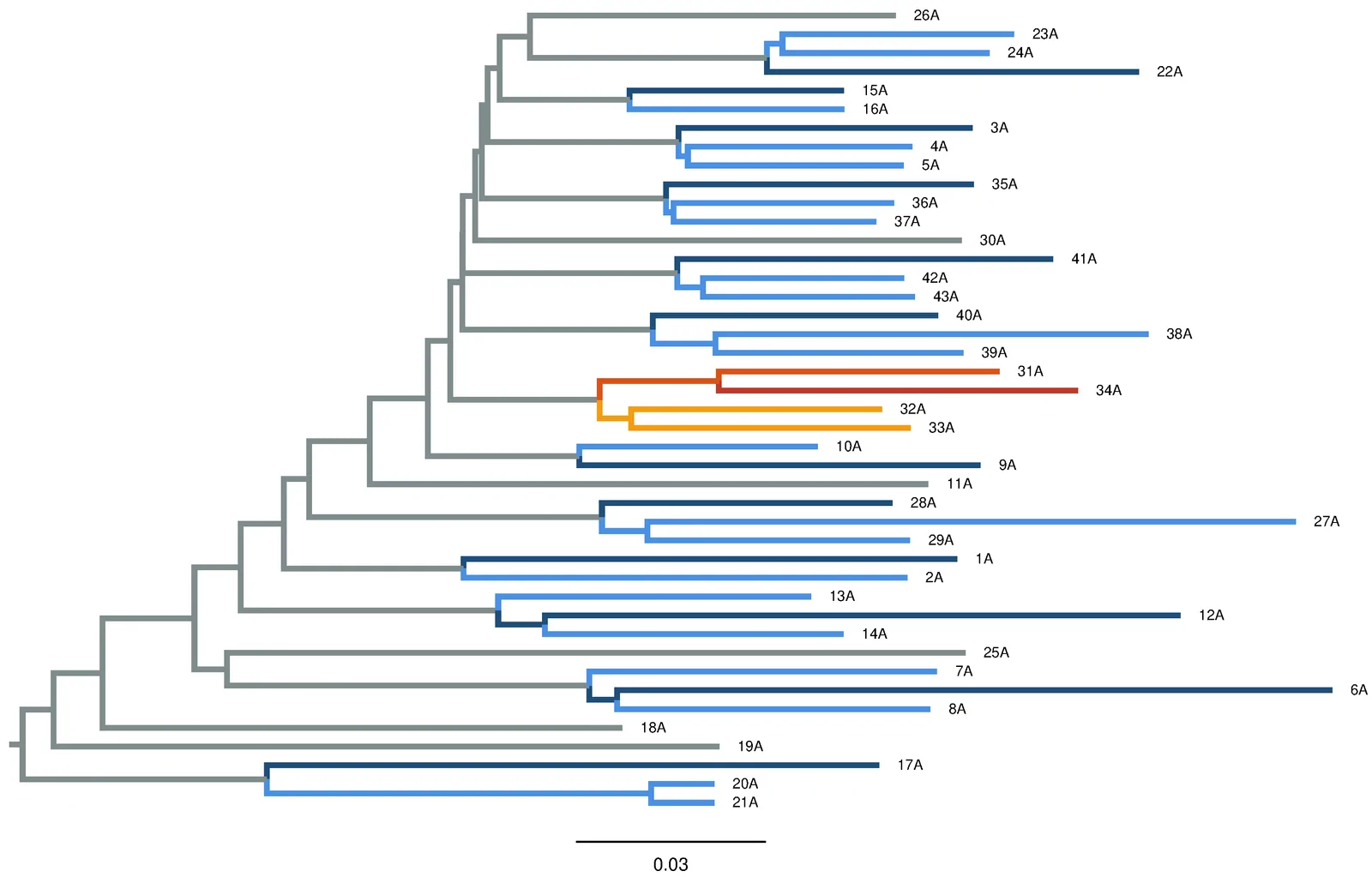
Neighbor-joining tree based on 654,363 LD-pruned genome-wide SNPs for 43 Simmental crossbred cattle. Known dam–calf groups are indicated by colored branches, with each color representing a distinct dam–calf group. The tree correctly recovered 12 of 13 true dam–calf groups (92.3%). The only ambiguous case involved twin sisters (31A and 34A) both clustering with the calves of 31A, but the dam–calf relationships were correctly identified.

The SNP-based NJ tree correctly recovered 12 of the 13 known dam–calf groups (92.3%, 95% CI: 64.0–99.8%) (Figure 3). The only ambiguity was observed in group 13, where the twin sisters 31A and 34A both clustered with the calves of 31A (32A and 33A), forming a single clade comprising the true dam, her twin sister and both calves. Although the true dam could not be uniquely distinguished from her twin sister, the dam–calf relationships were effectively preserved within the cluster.

### 3.3. Microsatellite-Based Pedigree Reconstruction

**Figure 4:**
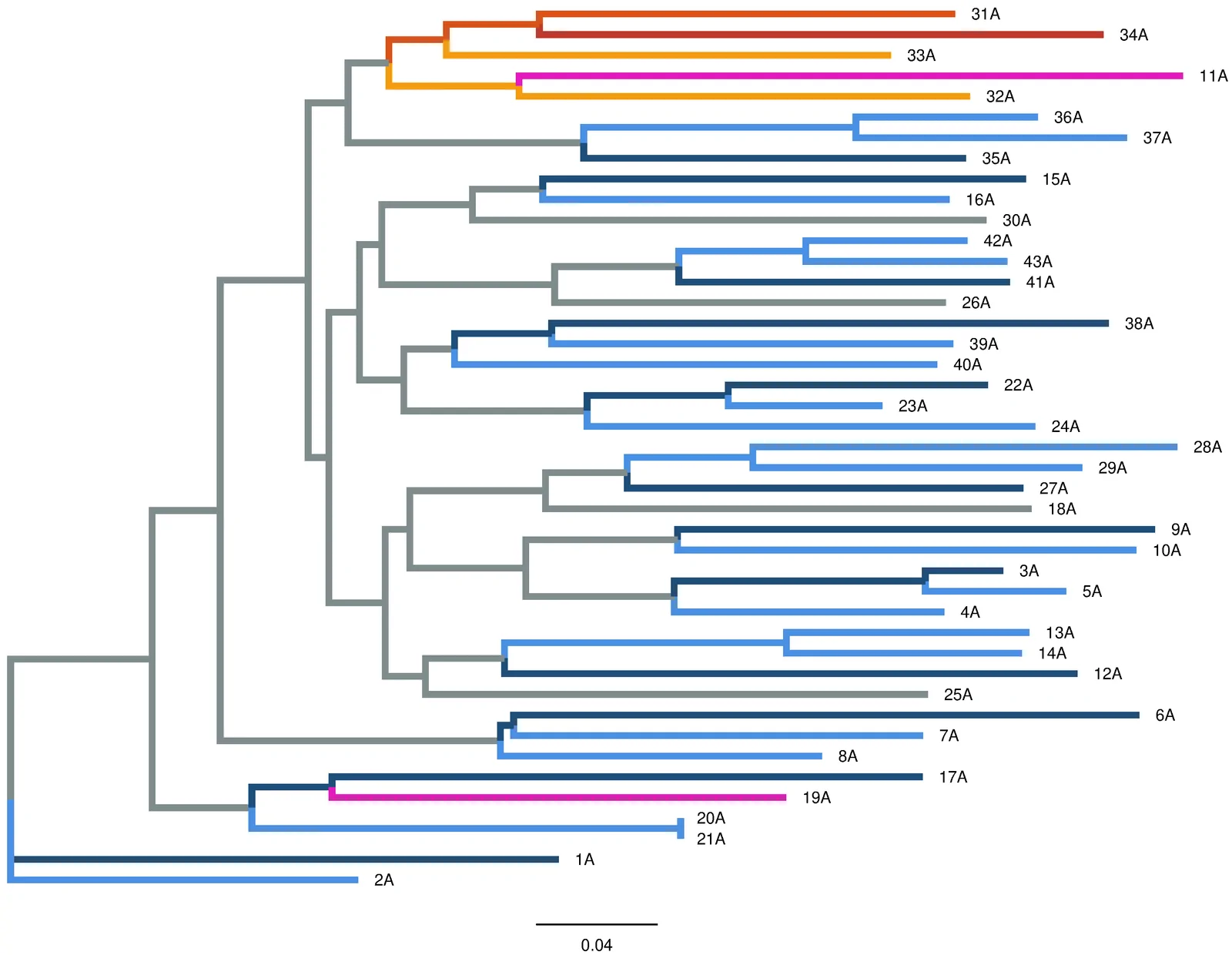
Neighbor-joining tree based on 12 ISAG microsatellite markers for the same 43 individuals. The tree correctly recovered only 11 of 13 dam–calf groups (84.6%). Two error types are marked in color: (i) false-positive clustering of 19A and 17A with calves 20A/21A, and (ii) complex misclustering of 31A, 34A, 11A, 32A and 33A.

Microsatellite markers yielded a lower recovery rate of 84.6% (11/13; 95% CI: 54.6–98.1%) (Figure 4). In one case, unassigned calf 19A clustered spuriously with dam 17A and her calves 20A and 21A, forming a false-positive cluster that obscured the true dam-offspring relationship. This pattern may reflect genotyping errors inherent to microsatellite data, such as allele dropout or the presence of null alleles [24]. In the other case, a more complex misclustering affected individuals 31A, 34A, 11A, 32A and 33A: calf 32A clustered most closely with the unrelated individual 11A, whereas its true dam 31A and maternal aunt 34A formed a separate sister clade with calf 33A. The complete dam–calf relationships for 31A and both twin calves were not accurately resolved, which likely reflects the limited resolution of microsatellite-based approaches [1].

### 3.4. Statistical Comparison and IBD Validation

The exact McNemar test showed no statistically significant difference between the recovery rates of the two methods (*p* = 1.0, two-sided). Across all 13 confirmed dam–calf groups (23 dam–calf pairs), the mean PI_HAT value was 0.482 (range: 0.394–0.601), consistent with first-degree relationships [25]. The SNP-based method correctly recovered 12 of these 13 groups. PI_HAT values between 34A and the calves of 31A were 0.2869 (with 32A) and 0.3213 (with 33A), both substantially lower than the expected value (0.5) for a true dam–calf relationship. IBD analysis also helped resolve the discrepancies observed in the microsatellite-based results. In the false-positive cluster involving individuals 19A and 17A, PI_HAT values between 19A and calves 20A and 21A were zero, confirming that 19A is completely unrelated to these calves. In the complex misclustering group (31A, 34A, 11A, 32A, 33A), the PI_HAT value between 11A and 32A was 0.0525, far below the expected 0.25 for half-sibling relationships [7,25].

## 4. Discussion

Our results demonstrate that genome-wide SNP data enable highly reliable pedigree reconstruction via NJ clustering in an admixed, twin-rich population with incomplete pedigree records, achieving a topological concordance of 92.3%. This extends previous findings on SNP-based parentage analysis in purebred or well-documented populations [19,20] to the more challenging scenario of smallholder crossbred herds. However, with only 13 dam–calf groups, the observed difference of one misclassified group did not reach statistical significance (exact McNemar *p* = 1.0), and the 95% confidence intervals for the two methods overlapped considerably (SNP: 64.0–99.8%; microsatellite: 54.6–98.1%). A larger sample size would be needed to establish a statistically reliable advantage of genome-wide SNPs.

Twin zygosity was a notable source of pedigree ambiguity in this herd, affecting both calf pairs and adult dams. The monozygotic twin pair (20A/21A) clustered correctly with their dam (17A), while the twin sisters 31A and 34A formed ambiguous clusters with calves 32A and 33A. We explicitly incorporated zygosity typing into our workflow, which has rarely been done in previous pedigree studies on twin cattle [1].

The standard ISAG microsatellite panel [16] was not uniformly informative in this population. Locus INRA023 was monomorphic (Polymorphism Information Content, PIC = 0), effectively reducing the number of informative markers to 11 [26]. This likely contributed to the lower recovery rate (84.6%) and the false-positive clustering involving individuals 17A and 19A. It should be noted that three calves (11A, 18A, and 19A) were excluded from their originally recorded dams based on preliminary microsatellite screening. In principle, this pre-screening step could introduce a bias, as the same marker type was later evaluated. However, the exclusion was based on clear multi-locus mismatches (2–5 loci) that are unlikely to represent false exclusions, and the microsatellite errors documented in this study were of a different nature (false-positive clustering rather than false exclusion). Our results suggest that in genetically diverse populations, the ISAG panel may require supplementation with more polymorphic markers [27].

It is also important to acknowledge that whole-genome resequencing at 12.7× coverage, as performed here, may not be economically feasible for routine parentage testing in smallholder farms. Lower-cost alternatives, such as the BovineSNP50 BeadChip or targeted SNP panels with a few hundred ancestry-informative markers, have been shown to provide sufficient resolution for parentage assignment in crossbred cattle populations [8]. Future work should evaluate the trade-off between cost and accuracy using such reduced SNP sets in twin-rich admixed herds. It should also be noted that the comparison between 12 microsatellites and thousands of SNPs is not an equivalent-marker comparison; rather, it reflects two different practical strategies. Future studies should systematically compare reduced SNP panels with marker numbers similar to the ISAG panel. Additionally, the reference populations used for admixture analysis were derived from publicly available datasets that may differ in sequencing depth and platform from the study samples. Although these differences can potentially affect PCA and ADMIXTURE results, the clear separation of lineages and the consistency across multiple analytical approaches suggest that the impact on our conclusions is limited.

Although twinning is relatively uncommon in most cattle populations, the broader challenge addressed here is pedigree reconstruction in admixed herds with incomplete farm records, which is common in smallholder systems.

Owing to the modest sample size, estimates of recovery rates may be subject to uncertainty. Parentage information was derived from farm records rather than a controlled pedigree, and the absence of sire and maternal-grandsire records limited further analysis. Nonetheless, our findings illustrate the practical utility of SNP-based methods in settings where pedigree records are incomplete or unreliable, particularly when combined with explicit zygosity typing.

## 5. Conclusions

Although the observed difference in recovery rates was not statistically significant due to the limited number of dam–calf groups, genome-wide SNPs showed a favorable trend in pedigree reconstruction accuracy compared with the standard ISAG microsatellite panel in this admixed, twin-rich population. The explicit determination of twin zygosity, which was integrated into the workflow, helped distinguish monozygotic from dizygotic twin pairs and identified an unexpected twin relationship among adult dams. When twin sisters remain unrecognized, offspring can be assigned to the wrong sister because both candidate dams cluster equally closely with the calf. Incorporating twin-typing into routine pedigree workflows therefore allows such ambiguity to be anticipated and flagged for additional verification, reducing the risk of incorrect dam–calf assignment, particularly in herds where twin births are common. The monomorphism of INRA023 further highlights the importance of selecting population-appropriate markers. For resource-limited settings, future work should evaluate lower-cost genotyping platforms, such as reduced SNP panels, to balance accuracy and affordability. Larger studies with more twin cohorts and optimized marker sets are warranted to confirm the trends observed here.

## Data Availability

The sequencing data that support the findings of this study have been deposited in the NCBI Sequence Read Archive (SRA) under BioProject accession number PRJNA1472885. The genomes of other cattle breeds used in this study are publicly available from GenBank.

## Supplementary Materials

The following supporting information can be downloaded at https://www.mdpi.com/article/10.3390/ani16172780/s1, Table S1: Sample information and parentage verification results for 43 Simmental crossbred cattle; Table S2: Primer information for the 12 microsatellite markers used in this study.

## Abbreviations

The following abbreviations are used in this manuscript:

ISAG	International Society for Animal Genetics
SNP	Single nucleotide polymorphism
IBS	Identity-by-state
IBD	Identity-by-descent
NJ	Neighbor-joining
PIC	Polymorphism information content
MAF	Minor allele frequency
LD	Linkage disequilibrium
PI_HAT	Proportion of identity-by-descent
