# Supplementary material for: Microsatellites Versus Genome-Wide SNPs Data for Pedigree Reconstruction in Twin Simmental Crossbred Cattle
Source: Animals (Basel). 2026 Sep 4;16(17):2780. doi: 10.3390/ani16172780 (PMC13564610; doi:10.3390/ani16172780)
Supplement: Supplementary file 1 [file animals-16-02780-s001.zip › animals-4467324-supplementary.pdf]

Table S1. Sample information and parentage verification results for 43 Simmental crossbred cattle.

ID	Category	Dam ID	Location	Verification status
1A	Dam	–	Yiyang	Confirmed dam
2A	Calf	1A	Yiyang	Confirmed (PI_HAT = 0.4196)
3A	Dam	–	Yiyang	Confirmed dam
4A	Calf	3A	Yiyang	Confirmed (PI_HAT = 0.5496)
5A	Calf	3A	Yiyang	Confirmed (PI_HAT = 0.5457)
6A	Dam	–	Yiyang	Confirmed dam
7A	Calf	6A	Yiyang	Confirmed (PI_HAT = 0.3938)
8A	Calf	6A	Yiyang	Confirmed (PI_HAT = 0.4050)
9A	Dam	–	Yiyang	Confirmed dam
10A	Calf	9A	Yiyang	Confirmed (PI_HAT = 0.5109)
11A	Calf	–	Yiyang	Excluded (5 MS mismatches)[a]
12A	Dam	–	Yiyang	Confirmed dam
13A	Calf	12A	Yiyang	Confirmed (PI_HAT = 0.4067)
14A	Calf	12A	Yiyang	Confirmed (PI_HAT = 0.3944)
15A	Dam	–	Yiyang	Confirmed dam
16A	Calf	15A	Yiyang	Confirmed (1 MS mismatch, PI_HAT = 0.6014)
17A	Dam	–	Yiyang	Confirmed dam
18A	Calf	–	Yiyang	Excluded (4 MS mismatches)[b]
19A	Calf	–	Yiyang	Excluded (2 MS mismatches)[b]
20A	Calf	17A	Yiyang	Confirmed (PI_HAT = 0.4091)
21A	Calf	17A	Yiyang	Confirmed (PI_HAT = 0.4096)
22A	Dam	–	Xumusuo	Confirmed dam
23A	Calf	22A	Xumusuo	Confirmed (PI_HAT = 0.5223)
24A	Calf	22A	Xumusuo	Confirmed (PI_HAT = 0.5153)
25A	Dam	–	Xumusuo	Background
26A	Dam	–	Xumusuo	Background
27A	Dam	–	Huanghua	Confirmed dam
28A	Calf	27A	Huanghua	Confirmed (PI_HAT = 0.3981)
29A	Calf	27A	Huanghua	Confirmed (PI_HAT = 0.4128)
30A	Dam	–	Kaihui	Background
31A	Dam	–	Gaoqiao	Confirmed dam (twin with 34A)
32A	Calf	31A	Gaoqiao	Confirmed (PI_HAT = 0.4945)
33A	Calf	31A	Gaoqiao	Confirmed (PI_HAT = 0.5376)
34A	Dam	–	Gaoqiao	Background (twin with 31A)
35A	Dam	–	Miluo	Confirmed dam
36A	Calf	35A	Miluo	Confirmed (PI_HAT = 0.5547)
37A	Calf	35A	Miluo	Confirmed (PI_HAT = 0.5578)
38A	Dam	–	Lianyuan	Confirmed dam
39A	Calf	38A	Lianyuan	Confirmed (PI_HAT = 0.5033)
40A	Calf	38A	Lianyuan	Confirmed (PI_HAT = 0.4849)
41A	Dam	–	Lianyuan	Confirmed dam
42A	Calf	41A	Lianyuan	Confirmed (1 MS mismatch, PI_HAT = 0.5021)
43A	Calf	41A	Lianyuan	Confirmed (PI_HAT = 0.5549)

[a] Excluded as offspring of 9A; retained as additional individual.

[b] Excluded as offspring of 17A; 18A and 19A were half-siblings (PI_HAT = 0.2625).

Table S2. Primer information for the 12 microsatellite markers used in this study.

**Locus**	**Primer sequence (5′–3′)**	**Fluorophore**	**Size (bp)**	**T_a (°C)**
BM1818	F: AGCTGGGAATATAACCAAAGG<br>R: AGTGCTTTCAAGGTCCATGC	FAM	253–272	58
BM1824	F: GAGCAAGGTGTTTTTCCAATC<br>R: CATTCTCCAACTGCTTCCTTG	HEX	178–192	58
BM2113	F: GCTGCCTTCTACCAAATACCC<br>R: CTTCCTGAGAGAAGCAACACC	FAM	123–143	58
TGLA53	F: GCTTTCAGAAATAGTTTGCATTCA<br>R: ATCTTCACATGATATTACAGCAGA	FAM	152–186	58
TGLA122	F: CCCTCCTCCAGGTAAATCAGC<br>R: AATCACATGGCAAATAAGTACATAC	VIC	137–181	58
TGLA126	F: CTAATTTAGAATGAGAGAGGCTTCT<br>R: TTGGTCTCTATTCTCTGAATATTCC	HEX	116–122	52
TGLA227	F: CGAATTCCAAATCTGTTAATTTGCT<br>R: ACAGACAGAAACTCAATGAAAGCA	FAM	76–102	52
ETH003	F: GAACCTGCCTCTCCTGCATTGG<br>R: ACTCTGCCTGTGGCCAAGTAGG	HEX	105–125	60
ETH10	F: GTTCAGGACTGGCCCTGCTAACA<br>R: CCTCCAGCCCACTTTCTCTTCTC	HEX	212–224	58
ETH225	F: GATCACCTTGCCACTATTTCCT<br>R: ACATGACAGCCAGCTGCTACT	HEX	141–159	55
INRA023	F: GAGTAGAGCTACAAGATAAAC<br>R: TAACTACAGGGTGTTAGATGAACTCA	FAM	190–220	58
SPS115	F: AAAGTGACACAACAGCTTCTCCAG<br>R: AACGCGTGTCCTAGTTTGGCTGTG	FAM	185–205	58

T_a: annealing temperature. F: forward primer; R: reverse primer.
